# Concomitant Xeroderma pigmentosum and disseminated small plaque psoriasis: first case of an antinomic association

**DOI:** 10.1186/1757-1626-1-74

**Published:** 2008-08-07

**Authors:** Khaled Ezzedine, Thierry Simonart, Michel Candaele, Denis Malvy, Michel Heenen

**Affiliations:** 1 Department of Dermatology, Hôpital Erasme, Université Libre de Bruxelles, 808, route de Lennik, Brussels, B-1070 Belgium; 2 Department of Internal Medicine and Infectious diseases, CHU St-André Bordeaux, 1, Rue Jean Burguet, 33075 France

## Abstract

We present the case of an eighteen-year-old Caucasian white boy who was diagnosed with xeroderma pigmentosum type A at age 5 and who experienced over the past year disseminated small plaque psoriasis confirmed with skin punch biopsy. The psoriatic lesions were successfully treated with multipotent topical corticosteroids and systemic retinoids. To our knowledge, the association between psoriasis and xeroderma pigmentosum has not been previously reported and may be regarded as unlikely when considering the pathogenesis of both diseases.

## Background

Xeroderma pigmentosum (XP) is a rare autosomal recessive disorder characterized by a defect of DNA-repair occurring during UV-induced damage. The disease is quite complex and different subsets of abnormalities in the DNA-repair system may be present during the course of the disease. Thus, patients with XP have a decreased cutaneous immune surveillance which results in an increased risk of UV-induced skin tumours at an early age. Basal cell carcinoma (BCC), squamous cell carcinoma (SCC), actinic keratoses, atypical moles and malignant melanoma, all associated with severe photoaging are commonly seen in such patients. The prognosis of XP is based on early diagnosis as to permit strict UV avoidance and early detection and treatment of skin tumours, especially in photo-exposed areas.

## Case presentation

**Figure 1:**
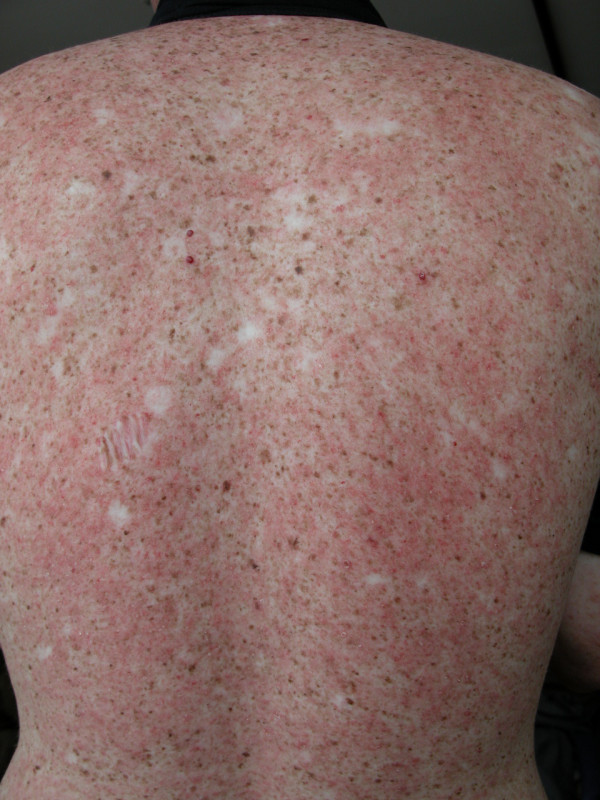
Association of small erythematous scaly plaques with multiple areas of hyperpigmentation resembling freckles on the patient's back.

**Figure 2:**
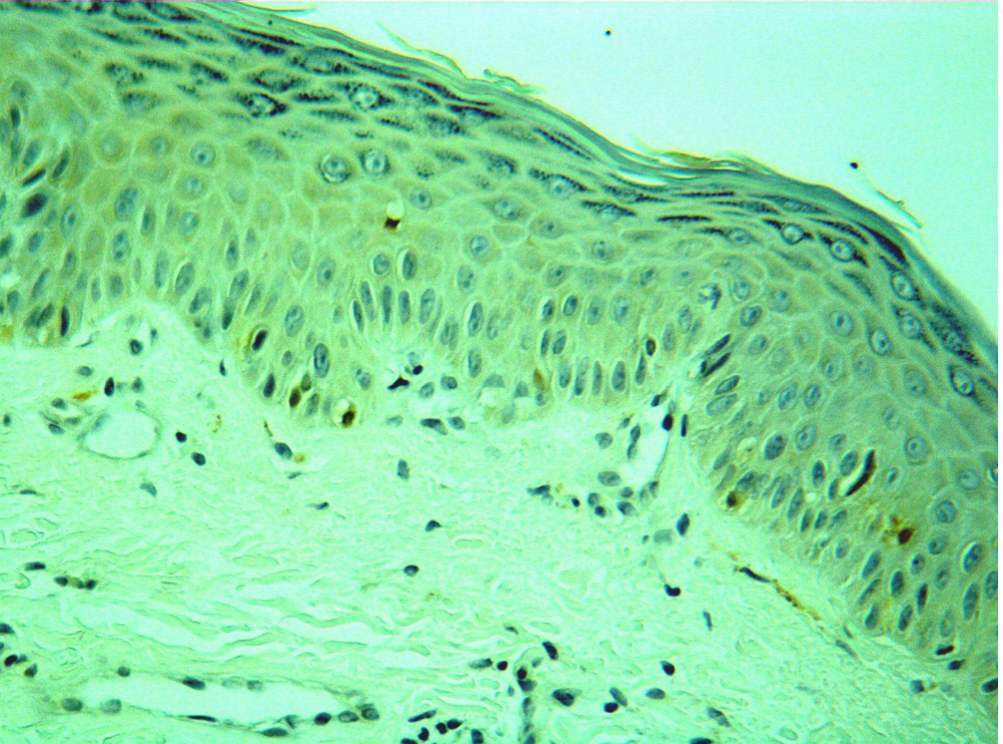
S100 staining showing reduced density of Langerhans cells in healthy skin.

We present the case of an eighteen-year-old white Caucasian boy who was diagnosed with XP type A at age 5. Other clinical signs include photophobia, keratitis and loss of eyelashes. Despite rigorous monitoring and photoprotection, the patient developed over 100 facial skin cancers (mainly BCC and SCC) treated with cryotherapy and surgery. Surprisingly, the patient experienced over the past year disseminated small plaque psoriasis (Fig. 1) confirmed with skin punch biopsy. S100 staining showed reduced density of Langerhans cells in healthy skin (Fig. 2), as previously reported in XP group A [1]. The psoriatic lesions were successfully treated with multipotent topical corticosteroids and systemic retinoids.

## Discussion

To our knowledge, the association between psoriasis and XP has not been previously reported and may be regarded as unlikely when considering the pathogenesis of both diseases.

XP patients exhibit Langerhans cell depletion, intensified impairment of natural killer (NK) cell function and enhanced UV-immunosuppression, possibly mediated through increased prostaglandin E2 production [1-4]. By opposition, psoriatic plaques are characterized by immune activation, with NK cell activation and decreased cyclo-oxygenase activity [5,6].

In addition, in XP, unrestricted cellular proliferation is associated with inactivation of members of the iNK4a/Arf locus, such as p14 and p16 [7]. By opposition, in psoriasis, members of the iNK4a/Arf locus are overexpressed, which may contribute to the senescent switch and resistance of psoriatic plaques to cellular transformation despite altered differentiation, angiogenesis, increased telomerase activity, proliferative changes and apoptosis resistance characterising psoriatic skin [8,9].

Further data are required to determine why and how those apparently antinomic diseases can coexist.

## Abbreviations

BCC: Basal cell carcinoma; DNA: desoxy ribonucleic acid; iNK: invariant natural killer; NK: natural killer; SCC: squamous cell carcinoma; UV: ultraviolet; XP: xeroderma pigmentosum.

## Competing interests

The authors declare that they have no competing interests.

## Authors' contributions

KE, TS, MH examined the patient. KE and TS were the major contributors in writing the manuscript. DM first seen the patient and helped to the redaction of the manuscript. MH and MC treated the patient and MH performed the histological examination for the skin biopsy. All authors read and approved the final manuscript.

## Consent

Written informed consent was obtained from the patient for publication of this case report and accompanying images. A copy of the written consent is available for review by the Editor-in-Chief of this journal.
